# A Broader Bioethics: *Topic Selection and the Impact of National Bioethics Commissions*

**DOI:** 10.1002/hast.713

**Published:** 2017-05-22

**Authors:** Jason L. Schwartz

## Abstract

*Comparative assessments of national bioethics commissions in the United States commonly look at the differences among these groups over their forty‐year history. A particular focus has been differences in the membership, mission, methods, and reports of the President's Council on Bioethics, which was active from 2001 until 2009, compared to those of its predecessors and the recent Presidential Commission for the Study of Bioethical Issues, active from 2009 until 2016. The differences are real, but disproportionate attention to them can obscure the substantial similarities in commissions’ structure and function throughout the history of expert bioethics advice to government. As the Trump administration considers what role, if any, a bioethics commission will play in its work, it would be well served to consider how choices regarding the design of such a group and the topics it examines can best facilitate the unique contributions it can make to the government and to the country*.

## Comparative Analyses

Comparative assessments of national bioethics commissions in the United States commonly look at the differences among these groups over their forty‐year history. A particular focus has been differences in the membership, mission, methods, and reports of the President's Council on Bioethics, which was active from 2001 until 2009, compared to those of its predecessors and the recent Presidential Commission for the Study of Bioethical Issues, active from 2009 until 2016. The differences are real, but disproportionate attention to them can obscure the substantial similarities in commissions’ structure and function throughout the history of expert bioethics advice to government. As the Trump administration considers what role, if any, a bioethics commission will play in its work, it would be well served to consider how choices regarding the design of such a group and the topics it examines can best facilitate the unique contributions it can make to the government and to the country.

The precise names of previous commissions have varied, as have the events that led to their establishment and the issues for which they are best remembered, yet the history of national bioethics advisory groups in the United States is fundamentally one of consistency. The earliest commissions of the 1970s were established by Congress, with more recent ones created by the president, but in all cases, the president or his designee has selected the members of each group.^1^ Their membership, generally twelve to fifteen at a time, has consistently represented a broad range of professional and disciplinary backgrounds in the humanities, social sciences, life sciences, law, and health professions. Even as bioethics has grown and matured as a field, members who identified themselves as “bioethicists” or viewed bioethics as a central professional or scholarly focus have made up a minority of commission membership.

All national bioethics commissions in the United States have been subject to the provisions of the Federal Advisory Committee Act, which requires regular review and justification by the executive branch of the need for each committee, balance among its membership with respect to demographics and geography, and, importantly, public access to its proceedings, materials, and deliberations. This includes the ability for members of the public to comment either in person or in writing on ongoing activities. While all prior bioethics commissions have complied with these baseline requirements, some have gone well beyond them by actively seeking public perspectives or otherwise engaging with groups outside of government as part of their work.^2^

Throughout their history, the working relationships between bioethics commissions and the executive branch have been consistent with an “arms‐length model” for interactions between outside expert advisors and their government sponsors.^3^ In this model, the government provides financial and administrative support for committee activities, responds to requests for information and perspectives, and often makes specific requests concerning topics to be examined. But once charged, groups of this type generally have considerable latitude with respect to how they structure their deliberations and recommendations. For all national bioethics commissions to date and other government advisory committees employing the arms‐length model, the case for the independence of their recommendations, both real and perceived, is strong—in as much as any government‐created and ‐funded group can make a claim to independence—but instances in which recommendations are ignored or rejected are rather common.

This contrasts with alternative designs for structuring relationships between outside advisors and the government—designs that, to date, have not been used for national bioethics commissions—in which the day‐to‐day relationships and interactions between government and expert advisors is one of cooperation or even collaboration.^4^ Closer relationships along these lines might facilitate the development of advice that reflects a more nuanced appreciation of the particular questions and challenges facing policy‐makers, but advice emerging from these alternative models is more susceptible to real and perceived threats to its independence.

A similar pattern of historical continuity emerges with respect to the subjects addressed by U.S. bioethics commissions. The topics that commissions are asked to study or choose to study are critical factors in the impact of their findings and recommendations. Two reports have had an indisputably transformative effect on public policy: the *Belmont Report*, from the National Commission for the Protection of Human Subjects of Biomedical and Behavioral Research (1974‐1978),^5^ and the report on the definition of death from the President's Commission for the Study of Ethical Problems in Medicine and Biomedical and Behavioral Research (1978‐1983).^6^ There have been numerous attempts to assess the impact of other commission reports, often through quantitative strategies such as counting citations in the academic literature, judicial opinions, and legislative proceedings—but such analyses, although they can be helpful, overlook other ways of defining and evaluating the roles that national bioethics commissions have had in science, medicine, health, and public policy.

For example, commissions have played an agenda‐setting role: they have identified or affirmed, explicitly or implicitly, issues for which ethical considerations are seen as most pressing or most relevant to government efforts in biomedicine or to bioethical inquiry in the many other settings where it occurs. U.S. bioethics commissions have focused their reports overwhelmingly on two topics (sometimes concurrently):^7^ one of these is biomedical—particularly clinical—research and the protection of human subjects, and the other is emerging biotechnologies, particularly those that have implications regarding the beginning or end of life.

This pattern is attributable in part to the actions and choices of government officials who have created and shaped previous bioethics commissions. Two groups—the National Commission of the 1970s and the Advisory Committee on Human Radiation Experiments (1994‐1995)—had mandates focused solely on human subjects research. The National Bioethics Advisory Commission (1996‐2001) was formally instructed upon its creation to consider the protection of human research subjects as one of its first priorities, and that group and its two successors were all asked to examine a novel biotechnology related to reproduction—human cloning, stem‐cell research, and synthetic biology, respectively—early in their tenures.

Most commissions, including these three most recent groups, have also been free to select topics on their own in addition to responding to any government requests. On occasion, commissions *have* focused substantially or entirely on issues other than biomedical research and emerging biotechnologies. The reports on ethics and Ebola from President Obama's commission, on caregiving for the elderly from President George W. Bush's council, and on securing access to health care from the President's Commission of the early 1980s are examples. But such reports are rare exceptions to a pattern of more than forty years.

As part of its 2016 meetings examining the past, present, and future of bioethics advice to government, the Presidential Commission for the Study of Bioethical Issues asked presenters to consider the overall impact of bioethics advisory bodies on health policy. How one defines “health policy” matters greatly in approaching this question. If we define the term broadly, so as to include policies related to all aspects of medical science, research, and practice that may affect human health, then the record of these groups is fairly robust. If, however, we define health policy in the manner that health policy departments, journals, and textbooks typically do—to refer, for example, to the organization, financing, and delivery of health care and its consequences for individual and population health—then the record is far more modest. We find almost nothing in the collected writings of these groups, particularly over the past two decades, on the ethical issues of health care access and affordability, health care quality, health disparities, mental health, pediatric health, environmental health, HIV/AIDS, disabilities, and any number of other issues in medicine and community, public, and population health.

The issues that bioethics commissions have studied and written on are surely important, and given the prominent federal role in oversight of human subjects research and biotechnology regulation and the long‐standing place of these areas in the history of bioethics, their consideration by outside advisors is certainly appropriate and valuable, whether the advice comes from national commissions; committees of the National Academies of Science, Engineering, and Medicine; or the Secretary's Advisory Committee on Human Research Protections.

But the time available to national bioethics commissions is likely their scarcest resource. Difficult choices are inevitable regarding how they direct their attention among many worthy priorities, and requests from a president or government agencies to study a specific area—often on an accelerated timetable—sometimes alters their plans. How and why specific topics were taken up by prior commissions require deeply context‐dependent and historically contingent answers, making retrospective critiques of why a particular topic was studied at a given moment instead of potential alternatives deeply problematic. Instead, a more useful approach is to consider the full body of work of national bioethics commissions in the United States over the past forty years and assess how well it reflects the range of issues for which highly visible bioethical analysis and advice could have been an asset to the president, the government, and the country.

Bioethics as a field has been criticized for its relative silence on topics beyond biomedical research and biotechnologies. If we accept the agenda‐setting function that bioethics commissions can play in bioethics writ large and the related role it has long been argued that commissions have played in its growth and development since the 1970s, then it is worth asking whether the pattern of topics that national bioethics commissions have studied merely reflects long‐standing patterns in the field or has in fact *contributed* to the field's gaps and omissions.

Leon Kass, chair of the President's Council on Bioethics, spoke often of his aspiration that his group would engage in what he described as a “richer bioethics,” one that “would feature careful and wisdom‐seeking reflection regarding the full range of human goods at stake in bioethical dilemmas.”^8^ What may hold greater promise for a future bioethics commission is a “broader bioethics,” one that directs its gaze not just to human subjects research and biotechnology but also toward the health care system and the health of communities and populations. A commission having a broader focus along these lines could speak to the ethical considerations embedded in the design of our health care system, the delivery of health care, and the promotion of public health. It could draw on and call attention to the small but growing body of work in bioethics on these subjects,^9^ and it might also be able to stimulate further work on these topics. And it would have considerable potential to promote ethically informed policy. Any future bioethics commission must zealously guard the independence of its deliberations and the integrity of its recommendations, all the more so presently given the threats to scientific inquiry and evidence‐based policy posed by the Trump administration. But doing so need not prevent future commissions from maintaining—even strengthening—the links between their work and the policy priorities of the administrations they serve.^10^

## References

[1] See the table in Alexander Capron's commentary in this report for a list of the commissions and some details about their establishment (A. M. Capron, “Building the Next Bioethics Commission,” *Goals and Practice of Public Bioethics: Reflections on National Bioethics Commissions*, special report, Hastings Center Report 47, no. 3 [2017]: S4‐S9, at S6‐S7). 28543661 10.1002/hast.710 PMC6617774

[2] J. L. Schwartz , “Democratic Deliberation in Bioethics,” presentation to the Presidential Commission for the Study of Bioethical Issues, Philadelphia, PA, May 27, 2015, https://bioethicsarchive.georgetown.edu/pcsbi/node/4943.html.

[3] J. L. Schwartz , “Reflecting on the Past, Present, and Future Impact of National Bioethics Advisory Bodies,” presentation to the Presidential Commission for the Study of Bioethical Issues, Washington, D.C., May 3, 2016, https://bioethicsarchive.georgetown.edu/pcsbi/node/5753.html.

[4] Ibid.

[5] National Commission for the Protection of Human Subjects of Biomedical and Behavioral Research, The Belmont Report: Ethical Principles and Guidelines for the Protection of Human Subjects of Research (1978; Washington, D.C.: U.S. Government Printing Office, 1979). 25951677

[6] President's Commission for the Study of Ethical Problems in Medicine and Biomedical and Behavioral Research, Defining Death: Medical, Legal and Ethical Issues in the Determination of Death (Washington, D.C.: U.S. Government Printing Office, 1981).

[7] Bioethics Research Library at Georgetown University, “U.S. Bioethics Commissions,” https://bioethics.georgetown.edu/library-materials/digital-collections/us-bioethics-commissions/.

[8] L. R. Kass , “Reflections on Public Bioethics: A View from the Trenches,” Kennedy Institute of Ethics Journal 15, no. 3 (2005): 221‐50, at 238. 16250106 10.1353/ken.2005.0018

[9] N. Daniels , “Equity and Population Health: Toward a Broader Bioethics Agenda,” Hastings Center Report 36, no. 4 (2006): 22‐35. 10.1353/hcr.2006.0058 16898358

[10] Schwartz, “Reflecting on the Past, Present, and Future Impact of National Bioethics Advisory Bodies.”

